# Altered gut microbiome plays an important role in AKI to CKD transition in aged mice

**DOI:** 10.3389/fmed.2023.1238960

**Published:** 2023-10-31

**Authors:** Myung-Gyu Kim, Won Yong Cho, Suk Min Chung, Young Eun Choi, Yina Fang, Myeong Soo Park, Sang Jun Park, Yoon Sook Ko, Hee Young Lee, Jihyun Yang, Se Won Oh, Sang-Kyung Jo

**Affiliations:** ^1^Division of Nephrology, Department of Internal Medicine, Korea University Anam Hospital, Seoul, Republic of Korea; ^2^Research Center, BIFIDO Co. Ltd., Hongcheon, Republic of Korea; ^3^Division of Nephrology, Department of Internal Medicine, Kangbuk Samsung Hospital, Seoul, Republic of Korea

**Keywords:** aging, acute kidney injury, chronic kidney disease, microbiome, chronic inflammation

## Abstract

**Introduction:**

This study investigated the role of renal-intestinal crosstalk in the transition from acute kidney injury (AKI) to chronic kidney disease (CKD) in elderly individuals.

**Methods:**

Using young and aged mice, we induced bilateral ischemia-reperfusion injury (IRI) and compared intestinal and kidney inflammation over 28 days. To determine the role of the microbiome in gut–kidney crosstalk, we analyzed the microbiome of fecal samples of the young vs. aged mice and examined the effects of probiotic supplementation.

**Results:**

In the post-IRI recovery phase, prolonged intestinal and renal inflammation along with dysbiosis were evident in aged vs. younger mice that was associated with severe renal dysfunction and fibrosis progression in aged mice. Probiotic supplementation with *Bifidobacterium bifidum BGN4* and *Bifidobacterium longum* BORI alleviated intestinal inflammation but not intestinal leakage, characterized by decreased inflammatory cytokine levels and decreased infiltration of macrophages, neutrophils, and Th17 cells. This was associated with improved M1-dominant renal inflammation and ultimately improved renal function and fibrosis, suggesting that renal–intestinal crosstalk in aged mice contributes to the transition from AKI to CKD.

**Discussion:**

Our study findings suggest that exacerbation of chronic inflammation through the gut–kidney axis might be an important mechanism in the transition from AKI to CKD in the elderly.

## Introduction

The incidence of acute kidney injury (AKI) is highest among elderly patients, and physiological aging, comorbid conditions, and environmental factors therein increase the prevalence of AKI (1, 2). AKI in the elderly is associated with an increased risk of mortality and progression to chronic kidney disease (CKD), including end-stage kidney disease (ESKD) (3–6). However, current treatments for AKI are supportive only; therefore, the increasing elderly population is highly susceptible to in-hospital mortality and morbidity.

Given the clinical burden of AKI in the elderly, elucidating the mechanisms of its exacerbation and progression to CKD is important in the development of new treatment strategies. Recent experimental studies reported that AKI in aged kidneys can accelerate renal fibrosis and progression to CKD associated with various pathological changes in the kidneys, such as tubular cell senescence, chronic inflammation, severe capillary rarefaction, and mitochondrial dysfunction (7–11). In particular, chronic inflammation of the aged kidney, characterized by persistent M1-dominant inflammation (10) or the presence of tertiary lymphoid tissue (11), is attracting attention as an important factor contributing to progression to CKD.

Interestingly, age-related changes in the gut environment are reported to be associated with age-related diseases through the exacerbation of chronic inflammation at the local and systemic levels (12, 13). Recent studies have demonstrated that, compared to young individuals, elderly individuals have increased intestinal permeability and decreased microbial diversity and beneficial microbe loads, and these changes lead to alterations in microbial metabolites and systemic chronic inflammation (14–16). Therefore, the gut may be an important gateway for chronic inflammation-induced geriatric diseases that possibly mediates CKD progression in elderly patients.

Gut–kidney crosstalk in AKI has been studied in several animal models. The administration of short-chain fatty acids (SCFA), important metabolites of microbiota, reduces local and systemic inflammation and improves renal dysfunction (17). Jo et al. reported that intestinal dysbiosis, inflammation, and leaky gut occur as a result of AKI and act as important determinants of its severity (18). However, little is known about the gut environment changes that occur with AKI in the elderly or their role in the transition from AKI to CKD.

Here we investigated the differences in the gut environment that occur in young vs. aged mice after an ischemia–reperfusion injury (IRI) and whether modulation of the gut microbiome could influence gut–kidney interactions or the AKI to CKD transition in aged mice.

## Materials and methods

### Experimental animals and renal IRI

Male C57BL/6 mice (weight, 20–25 g each) aged 6–8 weeks were purchased from Orient (Seongnam, Korea). Aged mice were up to 48 weeks old, and young mice were housed in the same facility as the aged mice for at least 3 weeks prior to experimentation. Four mice were housed in one cage and chow components were similar between the vendor (Charles-river 5 L79) and our animal facility (Safe-diet R3+) (Supplementary Table S1). All experimental protocols were approved by the Korea University Animal Protection Committee (IRB no KOREA-2021-0170) and followed the NIH publication “Principles for the Care of Laboratory Animals.” All mice had free access to water and food. Before IRI, mice were placed on a temperature-regulated pad, with monitoring to maintain their core body temperature at around 37°C. To induce the IRI, the mice were anesthetized by an intraperitoneal injection of 15 mg/kg ketamine and 2.5 mg/kg xylazine followed by bilateral renal pedicle clamping for 25 min. After the clamp was removed, renal reperfusion was observed for 1 min. The sham surgery was performed in a similar manner except for the renal pedicle clamping. For probiotic treatment, freeze-dried *Bifidobacterium bifidum* BGN4 (*B. bifidum* BGN4, BGN4) and *Bifidobacterium longum* BORI (*B. longum* BORI) powder (BIFIDO, Gangwon, Korea) were administered to the mice via oral gavage (2 × 10^9^ colony-forming units in 0.2 mL of sterile water) three times a week for 8 weeks. Serum creatinine levels were determined with a Beckman AU^®^ 5821 chemistry analyzer (Beckman Coulter, Brea, CA, United States) following the manufacturer’s instructions. Serum NGAL was measured using a mouse Lipocalin-2/NGAL DuoSet enzyme-linked immunosorbent assay kit from R&D Systems (Minneapolis, MI, United States).

### Flow cytometric analysis

Flow cytometric analyses of intestinal and lymph node cells were performed using fluorochrome-labeled monoclonal anti-CD4-APC (1:50, 17-0042), anti-IL17A-PE (1:50, 506904), anti-CD4-FITC (1:10, 11-0042), anti-CD25-APC (1:10, 17-0251) and anti-FoxP3-APC (1:10, 17-5773) antibodies purchased from Invitrogen (Carlsbad, CA, United States) or BioLegend (San Diego, CA, United States).

### Histological analysis

The histological analysis was performed using digital imaging and ImageJ software in a blinded manner. For the immunohistochemical staining, anti-mouse F4/80 (1:100, MCA497G, Serotec), Ly6G (1:200, 11-9668, eBioscience), CD80(1:500, ab254579, abcam) and CD206 (1:500, ab64693, abcam) were used. The quantification of staining was conducted in 8–10 randomly selected high-power fields (HPF) on each slide, followed by the analysis of either the mean number of positive cells or the extent of positive staining in the colon or renal cortex and outer medulla using Fiji ImageJ software (NIH, United States). The evaluation of all slides was conducted in a blinded fashion. Renal fibrosis was assessed using Masson’s trichrome–stained sections. To quantify the trichrome-stained area, we first selected a region of interest (including the cortex and outer medulla, excluding the blood vessels) and measured the total tissue area within it. The area of collagen fibrosis corresponding to the intense blue staining was measured and expressed as a percentage of the collagen area among the total tissue area.

### Intestinal permeability

The measurement of intestinal permeability was conducted using fluorescein isothiocyanate conjugated dextran (FITC-dextran, catalog number FD4; Sigma-Aldrich, St. Louis, MO). Following overnight water starvation, 44 mg/100 g of FITC-dextran dissolved in phosphate-buffered saline (PBS; 100 mg/mL) was orally administered, and FITC fluorescence activity in the blood was quantified after 4 h. This methodological procedure is based on the protocol outlined in Bio-protocol.^1^

### Real-time polymerase chain reaction

Real-time reverse transcription polymerase chain reaction was performed using an iCycler IQ real-time PCR detection system (Bio-Rad, Hercules, CA, United States) to detect tumor necrosis factor-α (TNF-α), inducible nitric oxide synthase (iNOS), interferon-γ (IFN-γ), interleukin (IL)-12, Arginase-1 (Arg-1), Foxp-3, transforming growth factor-β, and arginase-1 expression levels in the kidney and intestine. The reference gene used was 18 s rRNA (RT2 PCR Primer Set; Applied Biosystems, Foster City, CA, United States).

### Transdermal glomerular filtration rate measurement

Glomerular filtration rate (GFR) was measured non-invasively using a fluorometer (Medi-Beacon, St. Louis, MO, United States) to the mice and recording the transdermal fluorescence of fluorescein-isothiocyanate (FITC)-sinistrin (15 mg/kg; Fresenius-Kabi, Linz, Austria) over time. Once a fluorometer was attached to the shaved back of an anesthetized mouse, baseline fluorescence levels were recorded for 1 min and FITC-sinistrin was injected intravenously through the retroorbital sinus. The fluorometer was programmed to record transdermal measurements every 5 s. Measurements were taken for at least 1 h and stored in the device. GFR was calculated using a single-compartment model.

### Fecal microbiome analysis

Fecal pellets were gathered by cradling the mouse in one hand, allowing it to defecate directly into a sterile microtube held in the other hand. Alternatively, individual mice were placed in sterile beakers for a brief period, following which newly excreted fecal pellets were retrieved from the beakers.

Fecal pellets are stored at −70°C before undergoing total bacterial DNA isolation using the ZymoBIOMICS^™^ DNA Miniprep Kit (D4304, Zymo Research Corporation, Irvine, United States), following the manufacturer’s protocol. To amplify the 16S rRNA gene, the MiSeq 341F and MiSeq 805R primers were used in a PCR with Kapa HiFi Hot start Ready Mix DNA polymerase (Kapa Biosystems, Wilmington, MA, United States), followed by purification using the Agencourt AMPure XP kit (Beckman Coulter, Brea, CA, United States). The resulting library was quantified and size-estimated using the QIAxcel Advanced with QIAxcel DNA High Resolution Kit (QIAGEN, Hilden, Germany). Sequencing was performed using the Illumina MiSeq System (2 × 250 bp paired-end reads; Illumina, United States), in accordance with the Illumina 16S Metagenomic Sequencing Library preparation guide (Illumina, San Diego, CA, United States).

QIIME2 was used to process microbial sequences, including demultiplexing with q2-demux, denoising with DADA2, and taxonomic classification with the SILVA 132 database. Phylogenetic diversity was analyzed using Faith’s PD and observed features, while UniFrac distances were used to compare microbial community structure. The Kruskal-Wallis test and PERMANOVA were used to assess significant differences, and correlations were calculated with Spearman’s rank correlation coefficient in R. LEfSe was used to identify significant differences in bacterial abundance.

### Measurement of short chain fatty acids

The clear supernatant obtained from homogenized mouse fecal samples was analyzed for SCFAs using liquid chromatography tandem mass spectrometry in multiple reaction monitoring mode at the Seoul National University National Instrumentation Center for Environmental Management in Seoul, Korea.

### Statistical analysis

The results are presented as mean ± standard error, and statistical analyses were conducted using GraphPad Prism (version 9.0; GraphPad Software, San Diego, CA, United States). For comparison between two groups, an unpaired t-test was used, while differences in values among three or more groups were analyzed using one-way or two-way ANOVA, followed by Tukey post-test analysis. A *p*-value of less than 0.05 was considered statistically significant for all analyses.

## Results

### Persistent kidney inflammation after IRI in aged mice

**Figure 1:**
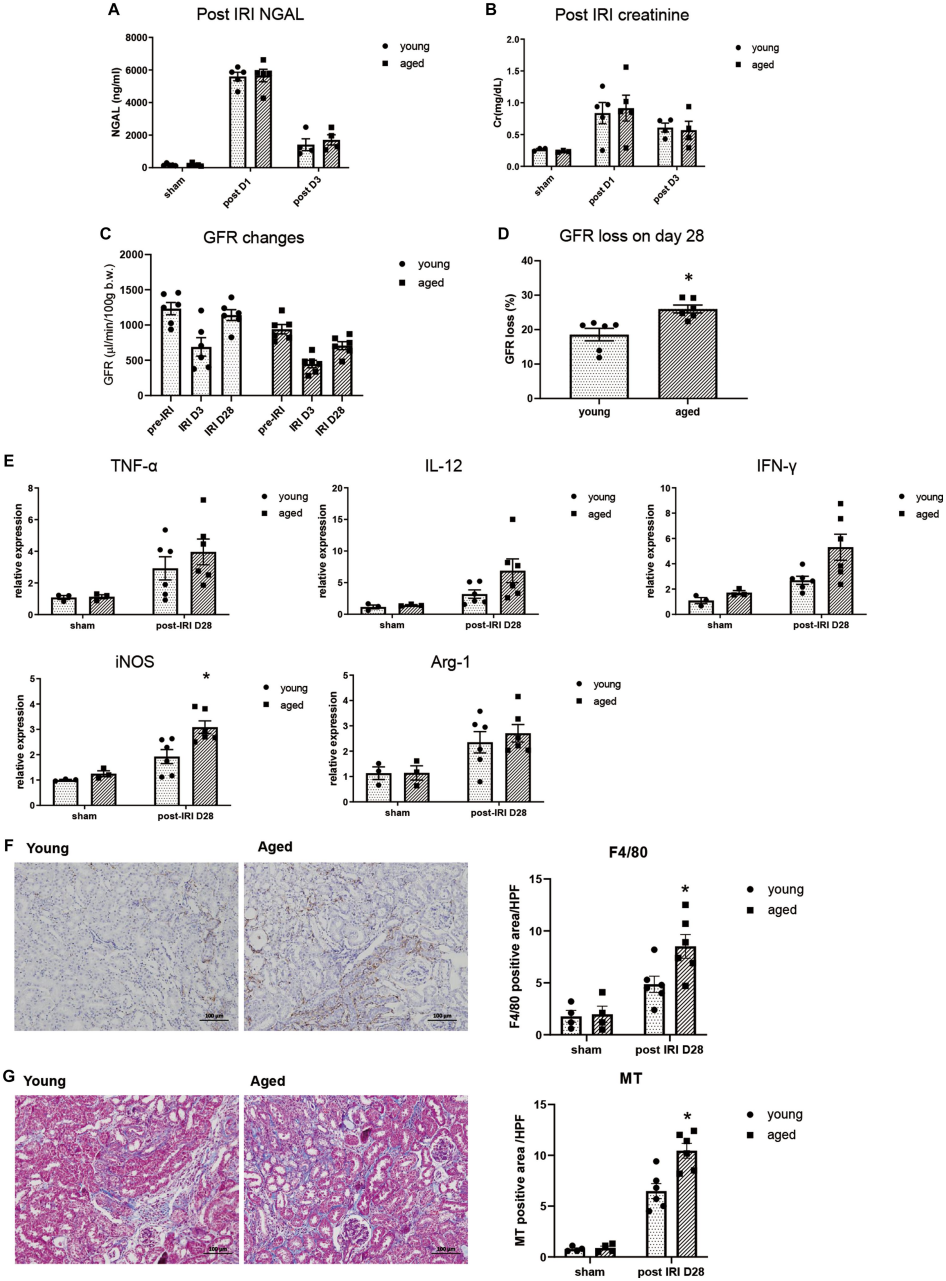
Kidney inflammation after IRI in young and aged mice. **(A–D)** Measured transdermal GFR using a fluorometer and serum NGAL and creatinine levels after IRI. GFR loss was calculated as [(baseline GFR - last GFR)/baseline GFR]. **(E)** mRNA expression of inflammation cytokines in kidney. **(F)** F4/80(+) inflammatory cell infiltration into the kidney (x100). **(G)** Masson’s trichrome (MT) staining of the kidney (x100). *p < 0.05 compared to young IRI mice.

We measured NGAL and creatinine on post-IRI days 1 and 3, and no significant difference was found between young and aged mice, suggesting that aging itself does not significantly affect AKI severity (Figures 1A,B).

The inflammatory response and CKD progression during the recovery phase after IRI were investigated in aged and young mice. After 4wk of IRI, infiltration of F4/80^+^ and CD80^+^ macrophages increased more than CD206^+^ macrophages, and increased proinflammatory cytokine, iNOS, but not Arg-1, was observed in the kidneys of aged vs. young mice, indicating persistent M1 predominant inflammation. This was accompanied by more fibrotic progression and loss of GFR in the aged mice (Figures 1C–G, Supplementary Figure S1).

### Young and aged microbiota alteration after IRI

**Figure 2:**
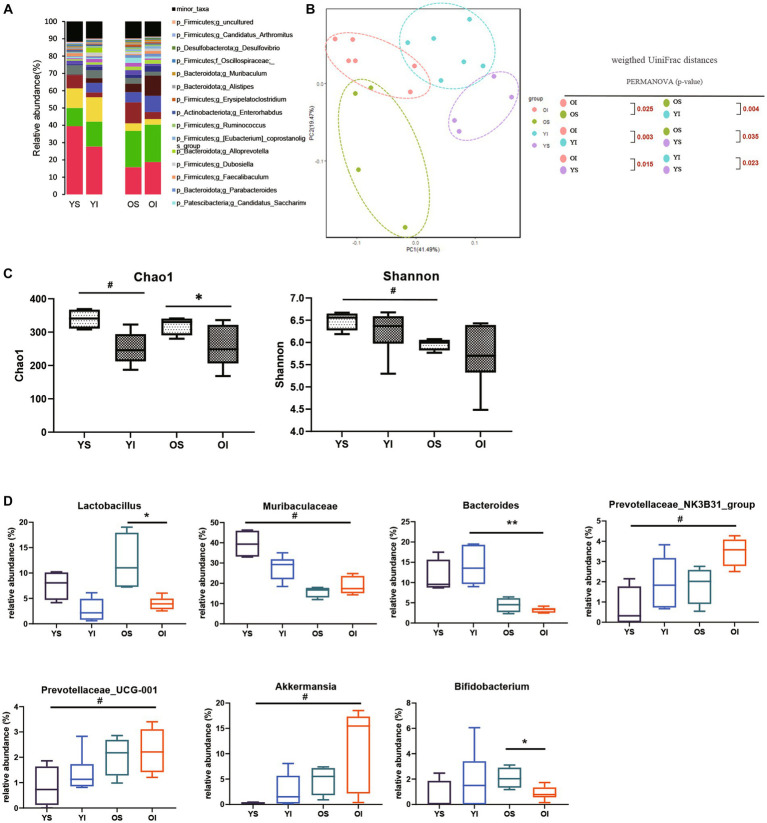
Microbiota alteration after IRI in young and aged mice. **(A)** Changes in microbial composition at genus level from stool sample in 4 groups. Only the genera with frequencies of >1% and with significant differences between the groups were included. **(B)** Principle coordinate analysis plot of weighted UniFrac distance. **(C)** Analysis of microbiome species richness (Chao1) and evenness (Shannon) in each group. **(D)** Relative abundances of specific microbiome. *p < 0.05 compared to old sham mice. **p < 0.05 compared to young IRI mice. ^#^*p* < 0.05 compared to young sham mice. YS, Young sham; YI, Young IRI; OS, Old sham; OI, Old IRI.

To assess the effect of aging on the kidney-gut axis concomitant with AKI, we first performed a 16S rRNA gene sequencing analysis of fecal samples from young and aged mice before and after IRI. Alpha diversity was decreased in aged vs. young mice, and richness was significantly decreased on post-IRI day 28 in both young and aged mice. In a principal coordinate analysis plot of weighted UniFrac distance, four groups formed clusters with statistically significant differences (Figures 2A–C). The microbiome abundance of aged IRI mice was characterized at the genus level by relative increases in *Prevotellaceae* and *Akkermansia* and relative decreases in *Muribaculaceae* and *Bacteroidetes* vs. the young sham or young IRI mice, and relative decrease in *Lactobacillus* and *Bifidobacterium* vs. the aged sham mice (Figure 2D, Supplementary Table S2).

### Intestinal environment during recovery phase after IRI in aged vs. young mice

In addition to the intestinal microbiome changes, inflammatory cytokine expression, intestinal barrier integrity, and histological changes in the colon were examined during the recovery phase after IRI in the aged vs. young mice.

**Figure 3:**
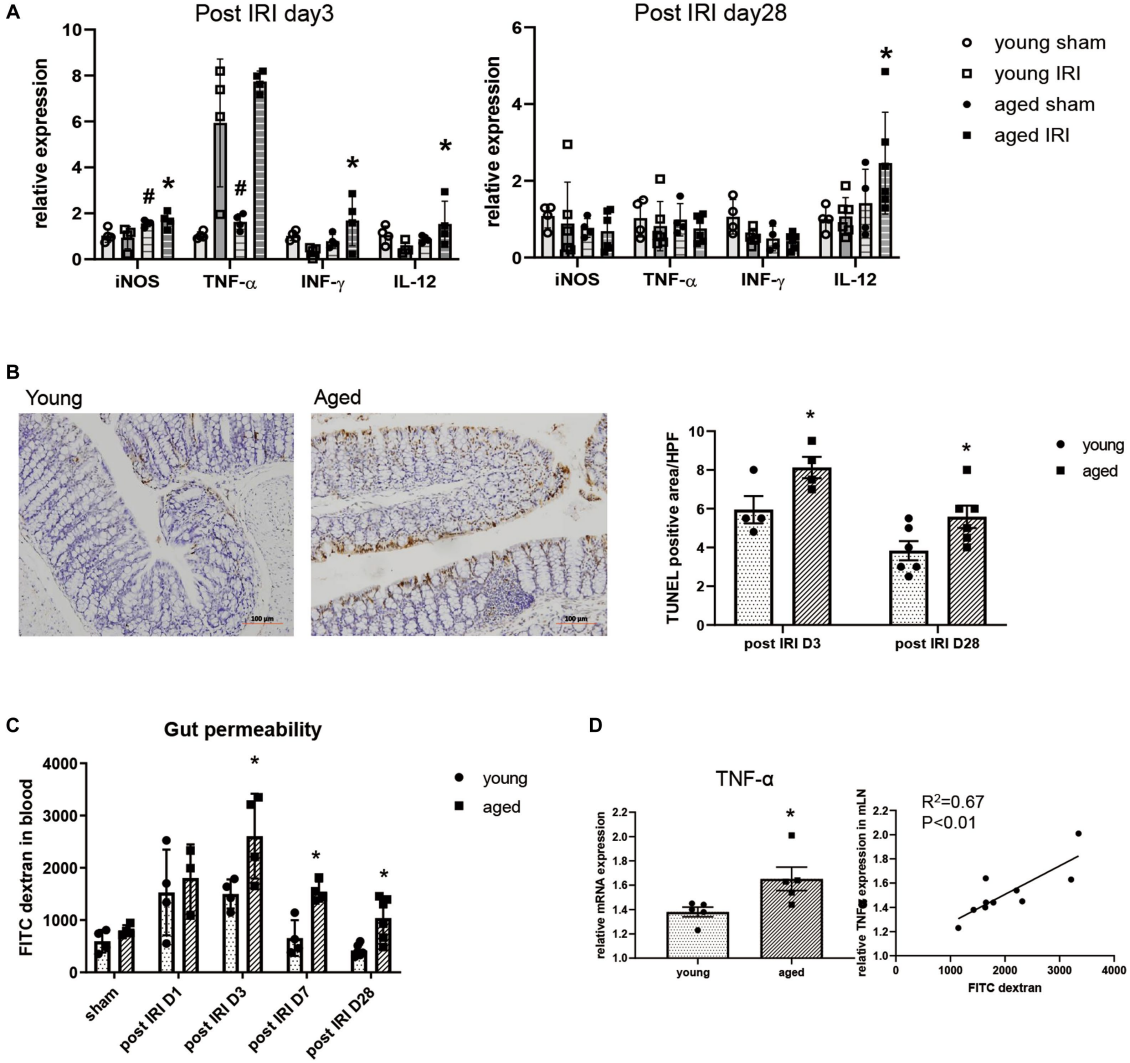
Change of intestinal environment during the recovery phase after IRI in young and aged mice. **(A)** mRNA expressions of inflammatory cytokines in colon day 3 and day 28 after IRI. **(B)** TUNEL (terminal deoxynucleotidyl transferase dUTP nick end labeling) positive apoptotic colon epithelial cells (x100). **(C)** Intestinal permeability by detecting fluorescein isothiocyanate conjugated (FITC)-dextran in blood after oral administration of FITC-dextran in day 1, 3, 7, and 28 after IRI. **(D)** Correlation between gut permeability and TNF-α mRNA expression in draining lymph node in post-IRI day 3. ^#^*p* < 0.05 compared to young sham, *p < 0.05 compared to young IRI mice.

Along with persistent M1 predominant inflammation in aged kidney, the expressions of inflammatory cytokines, such as iNOS, IFN-γ, and IL-12, were significantly higher in the colons of aged vs. young mice on day 3, while IL-12 mRNA expression remained elevated until day 28 (Figure 3A). Increased inflammatory cytokine levels in the colon were accompanied by increased numbers of TUNEL (terminal deoxynucleotidyl transferase dUTP nick end labeling) positive apoptotic colon epithelial cells and increased permeability of the colon in aged mice (Figures 3B,C). AKI-induced “leaky gut” on post-IRI day 3, showed a strong positive correlation with high TNF-α expression in draining lymph nodes (Figure 3D), suggesting a possible translocation of inflammatory mediators into the systemic circulation through disrupted intestinal barrier.

### Impact of probiotics on dysbiosis in old IRI

**Figure 4:**
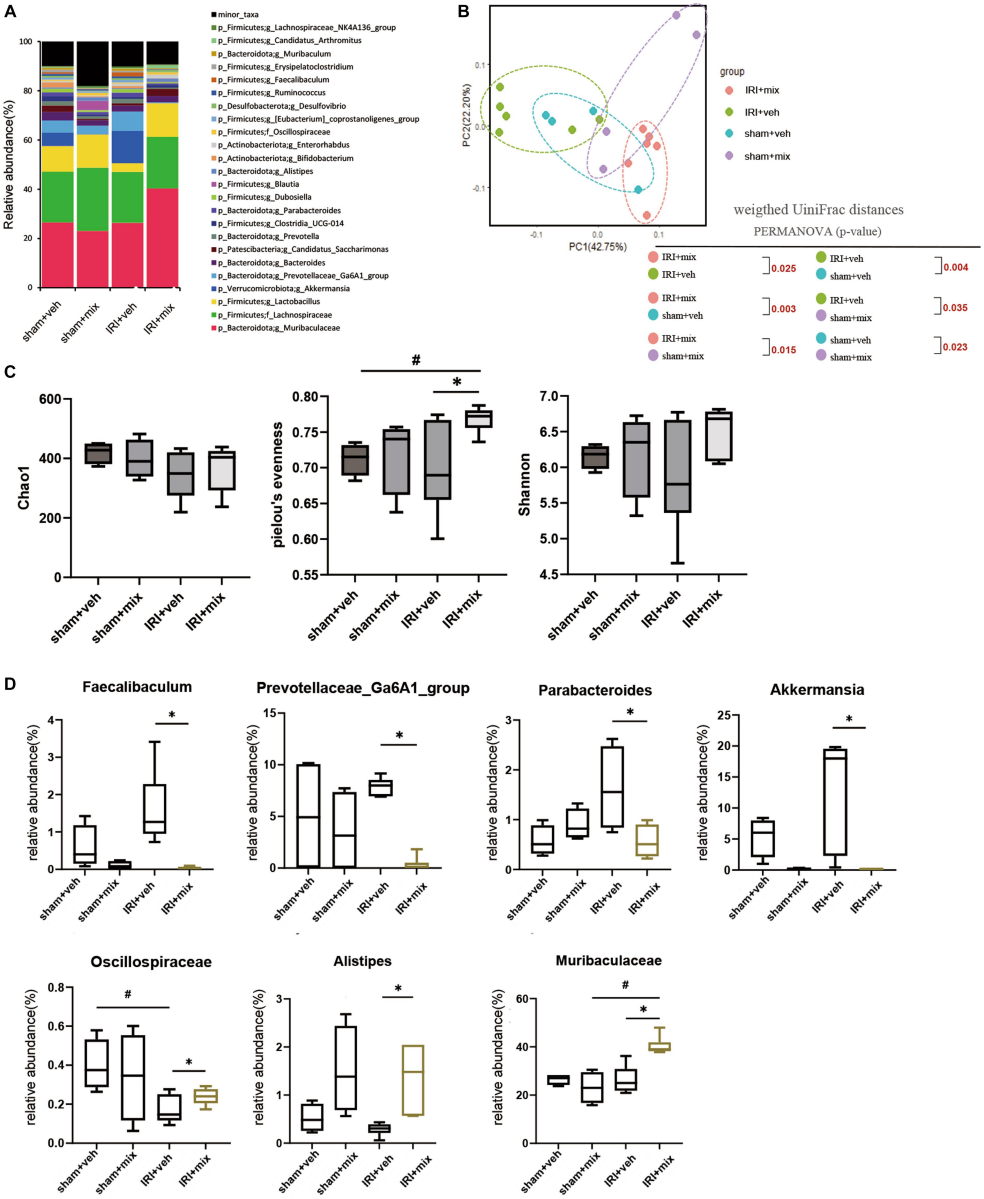
Microbial alteration after probiotics administration after IRI in aged mice. **(A)** Changes in microbial composition at genus level from stool sample in 3 groups. Only the genera with frequencies of >1% and with significant differences between the groups were included. **(B)** Principle coordinate analysis plot of weighted UniFrac distance. **(C)** Analysis of microbiome species richness (Chao1), Pielou’s evenness and diversity (Shannon) in each group. **(D)** Relative abundances of specific microbiome. ^#^*p* < 0.05 compared to sham+veh or sham+mix. *p < 0.05 compared to IRI + veh.

To evaluate the role of the microbiome on the gut–kidney axis in aged mice, we provided probiotics to the aged mice during the AKI to CKD transition and examined the changes in the gut microbiome. Treatment with combination BGN4 and BORI probiotics for 8 weeks induced changes in the bacterial composition in aged AKI mice (Figures 4A,B). The alpha diversity (evenness) in aged mice was improved by BGN4/BORI treatment (Figure 4C). We also found that BGN4/BORI treatment decreased the levels of *Prevotellaceae, Parabacteroides, Faecalibaculum and Akkermansia,* and increased the levels of *Alistipes, Muribaculaceae* and *Oscillospiraceae* vs. the vehicle group (Figure 4D).

### Impact of probiotics on gut inflammation after IRI

**Figure 5:**
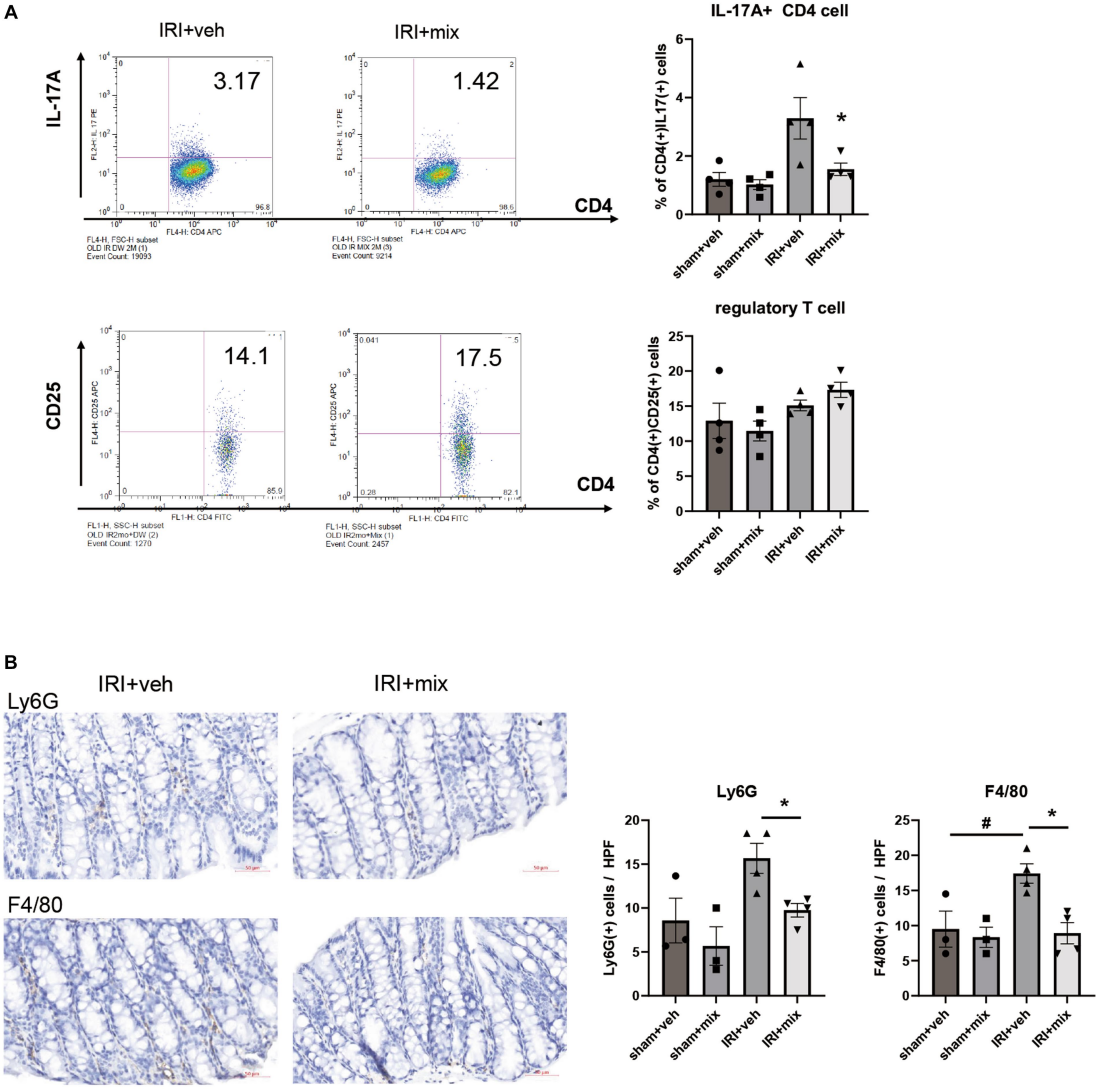

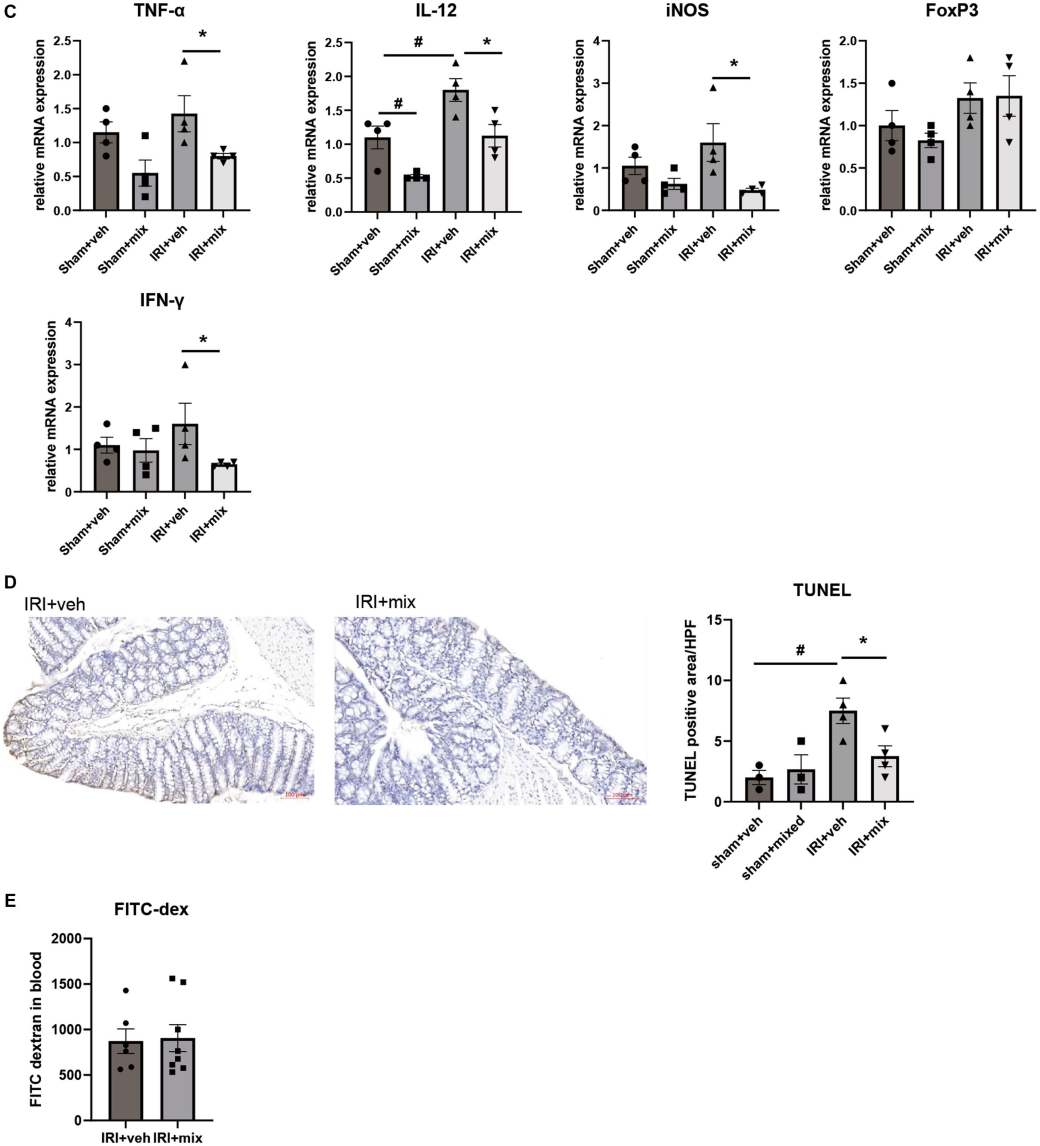
Effect of probiotics on intestinal inflammation after IRI in aged mice. **(A)** Flow cytometric detection of CD4+IL-17A+ T helper 17 cell in small intestine and CD4+CD25+ regulatory T cells in colon. **(B)** Representative images of infiltration of Ly6G+ neutrophils and F4/80+ macrophages in colon (x200). **(C)** mRNA expression of inflammatory cytokines in colon. **(D)** Representative images of colon TUNEL staining (x100). **(E)** Intestinal permeability by detecting fluorescein isothiocyanate conjugated (FITC)-dextran in blood after oral administration of FITC-dextran. ^#^*p* < 0.05 compared to sham+veh. *p < 0.05 compared to IRI+veh.

In addition to changes in microbial communities, BGN4/BORI treatment for 8 weeks after IRI resulted in a significant reduction in the CD4 + IL-17A+ Th17 cell population in the small intestine but no significant difference in the Treg population in the colon (Figure 5A). In addition, probiotic treatment significantly reduced the infiltration of colonic F4/80+ macrophages and Ly6G+ neutrophils along with decreased IL-12, iNOS, IFN-γ and TNF-α mRNA expressions in the colon (Figures 5B,C). In addition to the overall reduction in gut inflammation, BGN4/BORI administration improved intestinal epithelial apoptosis; however, no significant difference was noted in intestinal permeability between vehicle-and probiotic-treated mice (Figures 5D,E).

### Impact of probiotics on fecal SCFA and systemic inflammation

**Figure 6:**
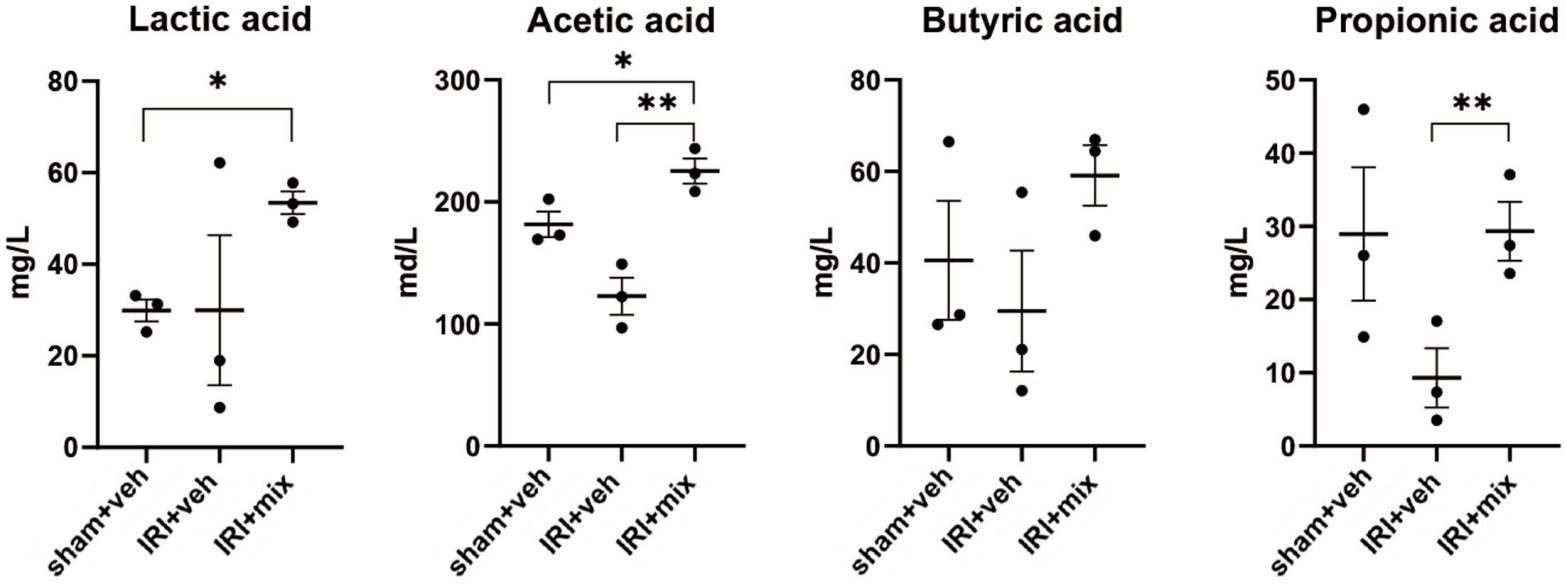
Impact of probiotics on fecal short chain fatty acid (SCFA) levels after IRI in aged mice. Tested SCFA level in fecal sample. *p < 0.05 compared to sham. **p < 0.05 compared to IRI+veh.

**Figure 7:**
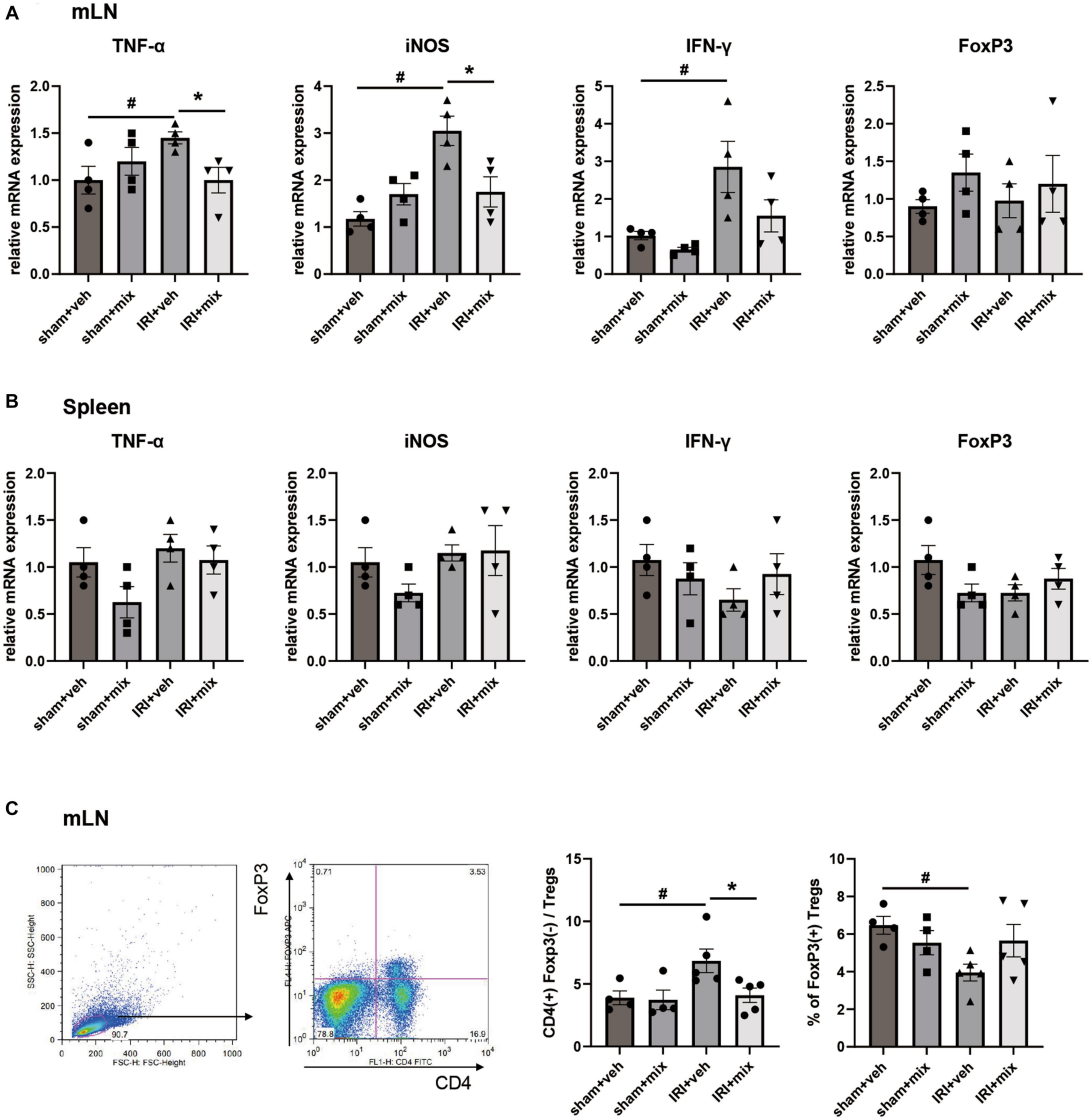
Impact of probiotics on systemic inflammation after IRI in aged mice. Comparison of mRNA expression of inflammatory cytokines and flow cytometric detection of CD4+T cells in **(A,C)** draining lymph node, **(B)** spleen. ^#^*p* < 0.05 compared to sham+veh. *p < 0.05 compared to IRI+veh.

SCFA, major bacterial products with anti-inflammatory properties, were tested in fecal samples from the mice. We found that the decreased acetic acid and propionic acid levels after IRI were significantly increased by BGN/BORI treatment (Figure 6). With changes in SCFA, levels of inflammatory cytokines such as iNOS and TNF-α, and the ratio of nonregulatory CD4 + T cells to Tregs decreased in the draining lymph node but not in the spleen (Figure 7).

### Impact of probiotics on kidney inflammation and outcomes

**Figure 8:**
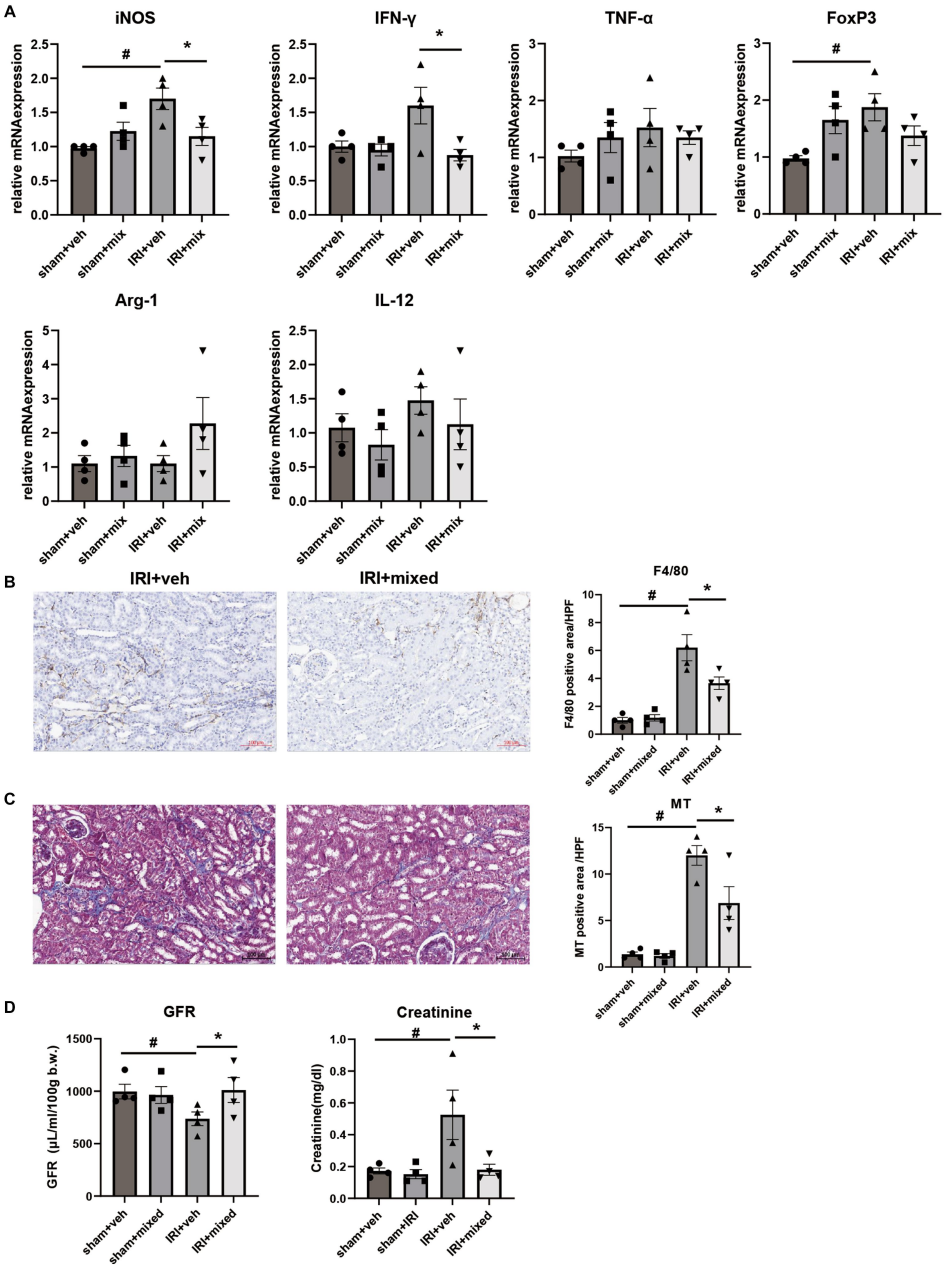
Impact of probiotics on kidney injury and inflammation after IRI in aged mice. **(A)** Comparison of mRNA expression of inflammatory cytokines in kidney. **(B)** Representative images of renal infiltration F4/80+ macrophages (x100) and **(C)** Masson’s trichrome (MT) staining of the kidney (x100). **(D)** Measured transdermal GFR using a fluorometer and serum creatinine level. ^#^*p* < 0.05 compared to sham+veh. *p < 0.05 compared to IRI+veh.

Improvement in intestinal inflammation was accompanied by a significant decrease in inflammatory cytokine and iNOS mRNA expression in the kidney. The histological analysis showed that F4/80+ cell renal infiltration was also significantly reduced after BGN4/BORI probiotic treatment, which suggests that improvement of gut and systemic inflammation through probiotic administration during the recovery phase ameliorated persistent M1 predominant inflammation in the aged kidney (Figures 8A,B). Finally, these changes in kidney inflammation led to improved renal function as well as improved renal fibrosis (Figures 8C,D). These results suggest that the gut–kidney axis may be an important therapeutic target in aged mice.

## Discussion

In the elderly, comorbid conditions, repeated exposure to nephrotoxic drugs or surgery, and structural and functional changes in aged kidneys all contribute to the high incidence and poor prognosis of AKI. Given the increase in life expectancy and the rapid expansion of the elderly population with a high prevalence of AKI, which often progresses to ESKD (1, 2), elucidating the role of aging in the transition from AKI to CKD is important for minimizing the risk of AKI in the elderly and developing novel therapeutic strategies.

Experimental studies have shown that various mechanisms are involved in the AKI to CKD transition, including mitochondrial dysfunction, capillary rarefaction, tubular cell arrest, and inflammation. In particular, chronic inflammation of the aged kidney is an important mechanism that accelerates the progression of AKI to CKD in the elderly. Sato et al. demonstrated the important role of tertiary lymphoid tissue and proinflammatory cytokine overproduction in aged mice. Treatment targeting tertiary lymphoid tissue formation using anti-CD4 monoclonal antibodies has the potential to ameliorate renal fibrosis and inflammation (11). Our recent study also demonstrated that kidneys from aged mice had persistent M1-dominant inflammation due to impaired M2 polarization caused by macrophage immunosenescence and interaction with senescent tubule cells. This M1 predominant inflammation in the aged kidney increased fibrosis progression and GFR loss after AKI (10).

The chronic inflammatory response in aged kidneys is also consistent with the persistent low-grade inflammation (called “inflammaging”) observed in various organs as an underlying mechanism of age-related diseases. Although the precise etiology of inflammaging and its contribution to disease exacerbation are largely unknown, recent evidence suggests that age-related dysbiosis of the gut microbiome may play an important role in the pathogenesis of systemic chronic inflammation in the elderly.

Clinical studies have reported significant differences in the microbial composition and gut environment of young vs. elderly individuals (14, 15). Decreased microbial diversity, reduced beneficial microbes, and reduced SCFA in the gut of the elderly can increase the gut permeability, leading to leakage of inflammatory bacterial components into the circulation and low-grade chronic inflammation. Floris et al. demonstrated that transferring aged microbiota to young germ-free mice promoted intestinal inflammation and leaky gut and increased T cell activation within the systemic compartments, indicating that the aged gut microbiota contributes to systemic inflammaging (13).

Several other studies reported a close relationship between the gut and kidney diseases. A recent report that AKI affects the intestinal microbiome, inflammation, and intestinal permeability, which also affects AKI outcomes, suggested that the bidirectional interaction of gut and kidney plays an important role in AKI (18). The occurrence of AKI in the elderly, in whom the intestinal environment is altered and chronic inflammation induced, may further change the gut microbiome and inflammatory response, which in turn may significantly affect AKI outcomes. From this perspective, we investigated whether changes in the gut environment accompanied by AKI in the elderly could play a role in the transition from AKI to CKD.

To clarify the gut–kidney axis in the elderly, we first examined the changes in the gut environment of IRI mice and found that AKI in aged mice exacerbated dysbiosis, which is characterized by decreased microbial richness and beneficial bacteria, and increased intestinal inflammation and permeability for a longer duration than in younger mice. The expression of inflammatory cytokines, such as iNOS, IFN-γ, and IL-12, was significantly higher in the colon of aged mice than in young mice, and IL-12 levels in particular were consistently increased during AKI to CKD transition, suggesting that IL-12 is an important mediator of IRI-induced intestinal inflammation in aged mice. The IL-12 cytokine family, such as IL-12 and IL-23, is known to play an important role in the development of Th1 and Th17 cells, and is essential for the induction of chronic intestinal inflammation mediated by innate or adaptive immune mechanisms, and blocking their signaling can reduce inflammation in experimental colitis models (19–21). Taken together, these alterations in the intestinal inflammatory milieu accompanying AKI and leaky gut may have exacerbated systemic and renal inflammation and ultimately fibrosis progression in aged mice.

The role of intestinal dysbiosis and inflammation in the transition from AKI to CKD in aged mice was determined by investigating whether post-IRI outcomes were altered by modulation of the microbiota with probiotics in aged mice. In particular, we evaluated the protective effects of BGN4 and BORI on the AKI to CKD transition.

In previous studies, the abundance of *Bifidobacterium* was inversely correlated with the degree of chronic inflammation in the elderly (22, 23), and probiotic treatment with *Bifidobacterium* prolonged the lifespan of mice by suppressing chronic low-grade inflammation of the colon (24, 25). BGN4/BORI treatment effectively improved age-related cognitive and memory deficits in aged mice, suggesting an anti-aging effect of these *Bifidobacterium* species (26).

In our data, *Bifidobacterium* was significantly decreased after IRI in aged mice but not in young mice, suggesting that reduction of *Bifidobacterium* may mediate aging-related inflammatory changes in intestine after IRI. We also observed that BGN4/BORI probiotics significantly altered the microbiome composition and improved the reduced diversity in AKI. Pathogenic bacteria, such as *Prevotellaceae* and *Parabacteroides* decreased, while some beneficial bacteria, such as *Alistipes, Muribaculaceae*, and *Oscillospiraceae* increased. These changes in the gut microbiota ameliorated intestinal inflammation and decreased the populations of intestinal F4/80+, Ly6G+, and Th17 cells. Although probiotics such as BGN4 were reported to affect Treg expansion in several studies (27, 28), no additional Treg expansion was observed in aged mice treated with BGN4/BORI in our study. The reasons for the discrepancy from previous results are unknown, but it was recently reported that although natural Tregs accumulate with aging in mice, extrathymic induction and differentiation of Foxp3 + regulatory T cells was impaired with age (29, 30).

Modulation of intestinal microbiota and inflammation was accompanied by an increase in fecal SCFA levels. An increase in bacteria such as *Alistipes, Muribaculaceae*, and *Oscillospiraceae* may have contributed to the increased production of SCFA in the intestine (31–33). SCFA are known to play an important role in maintaining epithelial barrier function by providing energy to commensal microbes and colonic epithelium. SCFA also reportedly promote immune tolerance by stimulating anti-inflammatory pathways in colonic macrophages and dendritic cells and increasing the Treg/Th17 ratio by interacting with G-protein-coupled receptors (34). SCFA can also move to the bloodstream and distant organs, such as the brain, lung, and kidney, and control systemic and local immune responses (35).

In this study, an increase in SCFA such as acetic acid and propionic acid in the feces may be associated with local and systemic anti-inflammatory effects, although no change in intestinal barrier function was noted in aged mice. It is unclear why improved intestinal inflammation and intestinal epithelial cell apoptosis did not ameliorate the intestinal leakage. Short-term microbial control alone may be unable to restore the increased intestinal permeability induced by chronic inflammation and the aging phenotypes of colonic epithelial cells.

Nevertheless, all of these changes in the intestinal environment led to the amelioration of persistent renal inflammation and improvement of fibrotic progression in aged mice with AKI, suggesting an important role of the gut–kidney axis in the transition from AKI to CKD in aged mice.

Despite these novel findings, our study had several limitations. First, we observed distinct dysbiosis in post-AKI aged mice but did not perform functional metagenomic studies; therefore, the role of changes in the gut microbiome could not be precisely identified. Second, although alterations in the gut microbiome composition by probiotic treatment ameliorated age-related renal inflammation, this does not indicate a causal relationship between gut and renal inflammation, and the underlying molecular mechanisms of the protective effects require further investigation. Third, although young mice were purchased at approximately 6 weeks of age and kept in the same facility as aged mice for at least 3 weeks prior to experiments, environmental influences from the supplier may have remained and influenced the results. Fourth, focusing only on *Bifidobacterium* presents certain limitations in adequately elucidating the distinction between young and aged mice. Within our data set, *Bifidobacterium* did not show significant differences between the two age groups. However, *Bifidobacterium* showed significant changes after IRI only in aged mice and is already recognized as an aging-associated microbiome. Nevertheless, an important limitation is the inability of individual *Bifidobacterium* species to comprehensively represent the diverse intestinal environment of aged mice.

Taken together, our results demonstrated that AKI in aged mice induced dysbiosis and prolonged intestinal and renal inflammation with additional fibrosis progression in the kidney and that BGN4/BORI treatment improved the AKI to CKD transition along with changes in the intestinal environment in aged mice. Therefore, these results suggest that the gut–kidney axis may be an important mechanism of AKI exacerbation in the elderly and may be a novel therapeutic target for aging-related renal disease.

## Data availability statement

The data presented in the study are deposited in the NCBI SRA (sequence Tead Archive) repository, accession number PRJNA1029352: https://www.ncbi.nlm.nih.gov/bioproject/PRJNA1029352.

## Ethics statement

The animal study was approved by Korea University Animal Protection Committee. The study was conducted in accordance with the local legislation and institutional requirements.

## Author contributions

M-GK: drafting the work. M-GK, WC, and S-KJ: substantial contributions to the conception or design of the work. M-GK, SC, YC, YF, MP, SP, YK, HL, JY, and SO: the acquisition, analysis, or interpretation of data for the work. All authors contributed to the article and approved the submitted version.

## Footnotes

^1^ http://www.bio-protocol.org/e1289
